# Association between smoking status and death from COVID-19 in South Korea: A nationwide cohort study

**DOI:** 10.18332/tid/168672

**Published:** 2023-07-24

**Authors:** Jae Woo Choi

**Affiliations:** 1 Community Care Research Center, Health Insurance Research Institute, National Health Insurance Service, Wonju, Republic of Korea

**Keywords:** smoking, COVID-19, Korean, nationwide cohort study

## Abstract

**INTRODUCTION:**

This study examined the association between smoking status and death from COVID-19.

**METHODS:**

This study used nationwide cohort data collected from the Korean National Health Insurance Service, linking to information on all individuals who tested positive for severe acute respiratory syndrome coronavirus 2 (SARS-CoV-2). The smoking status of subjects who participated twice in national health screenings between 2015 and 2018 was measured. This study investigated death from COVID-19 among those who tested positive from 1 January to 30 May 2020.

**RESULTS:**

This study included 4259 patients who tested positive for SARS-CoV-2 in Korea. After adjusting for all potential confounding factors, current smokers (adjusted odds ratio, AOR=3.75; 95% CI: 1.23–11.36) and recent quitters (AOR=3.74; 95% CI: 1.12–12.53) were associated with an increased risk of death from COVID-19 compared to never smokers. Compared with current smokers, long-term quitters (AOR=0.33; 95% CI: 0.11–0.95) and never smokers (AOR=0.27; 95% CI: 0.09–0.81) were associated with a reduced risk of death from COVID-19.

**CONCLUSIONS:**

Smoking was associated with an increased risk of death among patients with COVID-19. Given the lower possibility of death in long-term quitters with COVID-19, continuous smoking cessation among smokers or recent quitters is needed.

## INTRODUCTION

Globally, there were millions of confirmed cases of COVID-19, including more than 7 million deaths, according to the World Health Organization (WHO)^1^. Various risk factors for infection and severe outcomes of COVID-19 have been investigated^2,3^. Although smoking is a major cause of incident chronic diseases and premature death worldwide^4^, the effect of smoking status on COVID-19 outcomes is unclear. The low prevalence of current smoking among hospitalized patients with COVID-19 has been a consistent finding across most previous studies^5-7^. Previous studies demonstrated that past or current smoking was associated with higher odds of COVID-19 infection relative to never smoking^8,9^. A recent cohort study in England showed that former or current smokers were associated with a higher risk of death from COVID-19^10^. However, evidence for an association between smoking and death from COVID-19 has been mixed^10-13^.

The results from the meta-analysis were extracted from data on the ‘point prevalence’ of smoking status (i.e. assessment of smoking status at one particular time only). Since smoking behavior may change over time, repeatedly measuring information for smoking status within a specific period may be more reasonable^14^. Furthermore, the association between past smoking and death from COVID-19 may differ by smoking cessation period. Although evidence for the beneficial effect of smoking cessation on reducing the risk of death from COVID-19 may have significant implications during the COVID-19 pandemic, little is known about the association between smoking cessation and among patients with COVID-19. COVID-19 in Korea was primarily prevalent in specific regions (Daegu Metropolitan City, Gyeongsangbukdo) in the first half of 2020 and spread mainly in the metropolitan area and the whole country in the second half of 2020. During this study period, from January to May 2020, 11468 confirmed cases were reported. The distribution of characteristics such as sex and age among the study subjects included in this study was similar to that of the Korean population. This study investigated the association between smoking status and death from COVID-19 among patients with COVID-19 using nationwide cohort data.

## METHODS

### Data and participants

This study used data from the Korean National Health Insurance Service (NHIS), which is linked to information about the national health examination. The NHIS covers mandatory health insurance for all Korean citizens, and all insured individuals are required to have a standardized biennial health examination (an annual for manual workers). Since Korea has a single-payer national health system, the NHIS has all insured individuals’ demographic characteristics information (e.g. sex, age, household income, and residential area), results from national health screening (e.g. questionnaire survey on lifestyle, height, weight, and blood pressure measurements, and laboratory tests), all medical records regarding diagnoses, treatments, and prescribed drugs, and death information^15^. Korea Centers for Disease Control and Prevention (KCDC) database have epidemiological information for all individuals who underwent laboratory SARS-CoV-2 tests and the information for results of SARS-CoV-2 test in KCDC data is linked to the NHIS database^16^.

**Figure 1:**
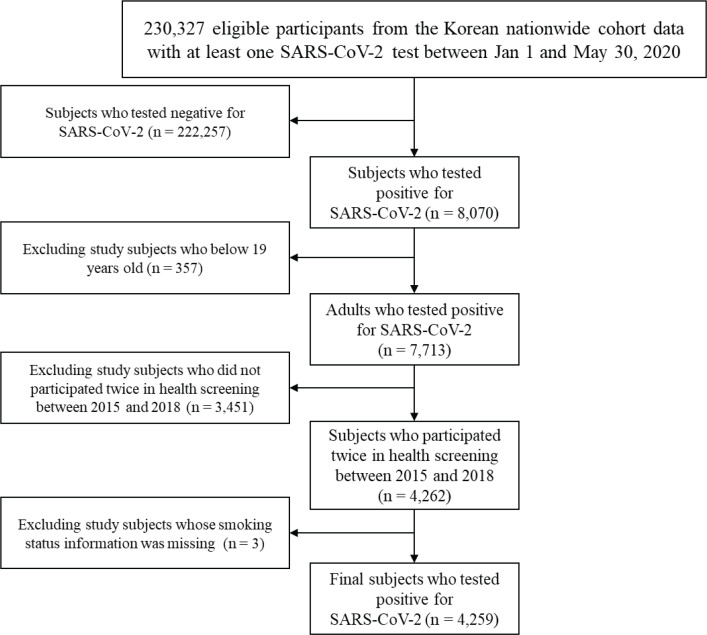
Flow chart of the study participants who were tested positive for SARS-CoV-2 from January to May 2020 in Korea

Among the 230327 individuals who tested positive for SARS-CoV-2 from 1 January to 30 May 2020, this study eliminated subjects who tested negative for SARS-CoV-2 (n=222257). This study also excluded participants aged ≤19 years (n=357) and eliminated study subjects who did not undergo a national health examination twice between 2015 and 2018 (n=3451). This study excluded subjects who had missing data for smoking status (n=3), and the final study sample included 4259 patients who tested positive for SARS-CoV-2 (Figure 1).

### Measures

The outcome of this study was death from COVID-19 among patients with COVID-19. The death information was ascertained from data of Statistics Korea and KCDC.

This study used information on smoking status surveyed from the self-reported questionnaire in the national health examination. The survey items regarding smoking status composed of current smoking, past smoking, and non-smoking. This study utilized information from the participants’ responses for two surveys on smoking status between 2015 and 2018 and study participants were classified into 4 groups: never smokers, recent quitters (≤2 years since smoking cessation), long-term quitters (>2 years since smoking cessation), and current smokers^17^.

Potentially confounding factors included sex, age, residential area, household income, body mass index (BMI), systolic blood pressure (SBP), diastolic blood pressure (DBP), fasting glucose, total cholesterol, estimated glomerular filtration rate (eGFR), and comorbidities. During the initial period in South Korea, there was an explosive outbreak of COVID-19 in the Daegu and Gyeongbuk regions^18^. Study participants were categorized into Daegu and Gyeongbuk regions and other regions. Household income was divided by the 5th quintile out of the total 20th quintile. BMI (kg/m^2^) was divided into groups as follows: 18.5 (underweight), 18.5–22.9 (normal weight), 23.0–24.9 (overweight), 25.0–29.9 (Class I obesity), and ≥30 kg/m^2^ (Class II obesity), based on the recommendation for Asian populations of the WHO^19^. Blood pressure was estimated once on the right arms of seated individuals who had rested for >5 minutes. Serum glucose levels and total cholesterol were measured by blood sampling after overnight fasting. eGFR was estimated by the Chronic Kidney Disease Epidemiology Collaboration equation for creatinine^20^. A history of hypertension (I10–15), diabetes mellitus (E10–14), dyslipidemia (E78), stroke (I60–63), ischemic heart disease (I20–25), chronic obstructive pulmonary disease (J43–J44, except J430), chronic kidney disease (N18), cancer (C00-97), asthma (J45–J46), chronic liver disease (K704, K711, K713-K715, K721, K73, K743, K767-K769), and mental illness (F00-99) were confirmed by the reporting of at least 2 claims within 1 year during the 3-year period before the COVID-19 test using the International Classification of Disease, Tenth Revision (ICD-10) code^21^.

### Statistical analysis

For patients with COVID-19, Pearson chi-squared test and ANOVA test were utilized to compare sociodemographic and clinical characteristics by smoking status. To explore the association between smoking status and the risk of death from COVID-19, this study used both current smoking and never smoking as the reference group, and multivariable logistic regression models with three incremental levels of adjustment as follows: Model 1: unadjusted; Model 2: adjusted for sex, age, household income, residential area, income level, and BMI; and Model 3: adjusted for all covariates.

All patient-related data were anonymized to ensure confidentiality. The study protocol was approved by the Institutional Review Board of Yonsei University (7001988-202006-HR-917-01E) and the requirements for informed consent were waived because the NHIS data were established following anonymization by guidelines for rigorous confidentiality.

## RESULTS

The general characteristics of study subjects who were tested positive for SARS-CoV-2 by smoking status are shown in Table 1. This study identified 309 current smokers, 236 recent quitters, 651 long-term quitters, and 3063 never smokers. The average SBP, DBP, fasting glucose, proportion of men, obesity, diabetes mellitus, hypertension, ischemic heart disease, stroke, chronic obstructive pulmonary disease, chronic kidney disease, and chronic liver disease among the current smokers were higher than those observed among the never smokers. Conversely, the average eGFR, the proportions of older adults (≥60 years), those living in Daegu and Gyeongbuk, low household income, dyslipidemia, cancer, asthma, and mental illness among the current smokers were lower than those among the never smokers. Table 2 indicated the results for the association between smoking status and the risk of death from COVID-19 among patients tested positive for SARS-CoV-2. After adjusting for all potential confounding factors, current smokers (AOR=3.75; 95% CI: 1.23–11.36) and recent quitters (AOR=3.74; 95% CI: 1.12–12.53) were associated with an increased risk of death from COVID-19 compared to never smokers. There was no significant association between long-term quitters (AOR=1.22; 95% CI: 0.42–3.54) and death from COVID-19. Compared with current smokers, long-term quitters (AOR=0.33; 95% CI: 0.11–0.95) and never smokers (OR=0.27; 95% CI: 0.09–0.81) were associated with a reduced risk of death from COVID-19. There was no significant association between recent quitters (AOR=1.00; 95% CI: 0.30–3.35) and death from COVID-19.

**Table 1:** General characteristics of Korean patients who were tested positive for SARS-CoV-2 from January to May 2020, by smoking status (N=4259)

*Characteristics*	*Total n*	*Smoking status*								*p*
		*Current smokers*		*Recent quitters*		*Long-term quitters*		*Never smokers*		
		*n*	*%*	*n*	*%*	*n*	*%*	*n*	*%*	
**Total**	4259	309	7.3	236	5.5	651	15.3	3063	71.9	
**Men**	1808	289	93.5	205	86.9	621	95.4	693	22.6	<0.001
**Age** (years)										
20–59	2786	232	75.1	146	61.9	370	56.8	2038	66.5	<0.001
≥60	1473	77	24.9	90	38.1	281	43.2	1025	33.5	
**Residential area**										0.002
Daegu and Gyeongbuk	2637	165	53.4	134	56.8	396	60.8	1942	63.4	
Other regions	1622	144	46.6	102	43.2	255	39.2	1121	36.6	
**Household income** (quantile)										<0.001
First (lowest)	1086	61	19.7	48	20.3	121	18.6	856	27.9	
Second	634	29	9.4	24	10.2	63	9.7	518	16.9	
Third	740	44	14.2	48	20.3	101	15.5	547	17.9	
Fourth	804	72	23.3	53	22.5	135	20.7	544	17.8	
Fifth (highest)	995	103	33.3	63	26.7	231	35.5	598	19.5	
**BMI** (kg/m^2^)										<0.001
<18.5	134	4	1.3	4	1.7	6	0.9	120	3.9	
18.5–22.9	1476	74	23.9	74	31.4	150	23.0	1178	38.5	
23–24.9	1072	89	28.8	57	24.2	166	25.5	760	24.8	
25–29.9	1372	117	37.9	88	37.3	289	44.4	878	28.7	
≥30	204	24	7.8	13	5.5	40	6.1	127	4.1	
**Comorbidities**										
Diabetes mellitus	830	70	22.7	56	23.7	174	26.7	530	17.3	<0.001
Hypertension	1115	79	25.6	73	30.9	229	35.2	734	24.0	<0.001
Dyslipidemia	1635	106	34.3	99	41.9	287	44.1	1143	37.3	0.003
Ischemic heart disease	228	22	7.1	18	7.6	57	8.8	131	4.3	<0.001
Stroke	105	9	2.9	4	1.7	22	3.4	70	2.3	0.319
Chronic obstructive pulmonary disease	75	14	4.5	6	2.5	24	3.7	31	1.0	<0.001
Chronic kidney disease	38	4	1.3	7	3.0	11	1.7	16	0.5	<0.001
Cancer	226	11	3.6	13	5.5	41	6.3	161	5.3	0.364
Asthma	576	29	9.4	51	21.6	113	17.4	383	12.5	<0.001
Mental illness	1101	63	20.4	58	24.6	171	26.3	809	26.4	0.134
Chronic liver disease	1019	71	23.0	61	25.8	203	31.2	684	22.3	<0.001
SBP (mmHg), mean ± SD	121.0 ± 14.9	122	12.8	124	13.9	126	14.3	120	15.0	<0.001
DBP (mmHg), mean ± SD	75.0 ± 9.8	76	9.2	78	9.6	78	10.0	74	9.7	<0.001
Fasting glucose (mg/dL), mean ± SD	101.0 ± 25.8	108	37.4	105	26.9	105	25.1	99	24.1	<0.001
Total cholesterol (mg/dL), mean ± SD	193.7 ± 38.9	196	43.5	188	40.5	185	39.1	196	38.1	<0.001
eGFR (mL/min/1.73m^2^), mean ± SD	90.5 ± 21.5	90	19.8	88	20.4	86	17.4	92	22.4	<0.001

SD: standard deviation. BMI: body mass index. SBP: systolic blood pressure. DBP: diastolic blood pressure. eGFR: estimated glomerular filtration rate.

**Table 2:** Association between smoking status and risk of Death from COVID-19 among Korean patients tested positive for SARS-CoV-2 from January to May 2020 (N=4259)

*Variable*	*Number of study subjects*	*Cases*	*Model 1*		*Model 2*		*Model 3*	
			*OR*	*95% CI*	*AOR*	*95% CI*	*AOR*	*95 % CI*
**Smoking status** (Ref. never smokers)								
Never smokers	3063	39	1.00		1.00		1.00	
Recent quitters	236	13	4.52	1.95–10.48	2.12	0.88–5.10	3.74	1.12–12.53
Long-term quitters	651	32	4.01	1.94–8.25	1.56	0.73–3.33	1.22	0.42–3.54
Current smokers	309	10	2.59	1.28–5.24	1.65	0.75–3.65	3.75	1.23–11.36
**Smoking status** (Ref. current smokers)								
Current smokers	309	10	1.00		1.00		1.00	
Recent quitters	236	13	1.74	0.75–4.05	1.28	0.53–3.09	1.00	0.30–3.35
Long-term quitters	651	32	1.55	0.75–3.19	0.95	0.45–2.02	0.33	0.11–0.95
Never smokers	3063	39	0.39	0.19–0.78	0.61	0.27–1.34	0.27	0.09–0.81

Model 1: unadjusted. Model 2: adjusted for sex, age, household income, residential area, and body mass index. Model 3: all covariates in Model 2 plus SBP, DBP, fasting glucose, total cholesterol, eGFR, and comorbidities. AOR: adjusted odds ratio. SBP: systolic blood pressure. DBP: diastolic blood pressure. eGFR: estimated glomerular filtration rate.

## DISCUSSION

This nationwide cohort study investigated association between smoking status and death from COVID-19 among patients with COVID-19. This study found that current smokers and recent quitters were associated with an increased risk of death from COVID-19 compared to never smokers. Compared with current smokers, long-term quitters, and never smokers were associated with reduced risk of death from COVID-19. This study found that current smokers and recent quitters were associated with an increased risk of death from COVID-19 compared to never smokers. Simons et al.^11^ found that past smokers compared with never smokers were at increased risk of mortality among patients having COVID-19, whereas current smokers were not associated with risk of mortality. However, Patanavanich and Glantz^12^ showed that smoking was associated with an increased risk of death from COVID-19. Both of these studies are meta-analysis studies and methodological differences such as inclusion criteria of research data may attribute to conflicting results. Williamson et al.^13^, reporting from a large UK study, showed a decreased risk for death in current smokers compared with never smokers when fully adjusted, possibly indicating a Table 2 Fallacy and suggested a need to be clarified as the epidemic progresses and more data accumulate. The biological and inflammatory cascade that occurs upon SARS-CoV-2 infection may be particularly devastating for smokers^22^. Considering that significant association between recent quitters, but not long-term quitters, and death from COVID-19, there seems to be a possibility that the negative factors of smoking accumulated recently may be associated to fatal outcomes, and further research is needed.

### Limitations

This study has several limitations. First, this study may include potential selection bias because it included subjects who underwent two national health examinations between 2015 and 2018, thus approximately 55% of all adults who tested positive for SARS-CoV-2 between 1 January and 30 May 2020. Second, information on smoking status was collected from a self-reported questionnaire, which is subject to recall and reporting bias. Since smoking status also changes over time, the smoking status measured in this study may differ from that at the time of the SARS-CoV-2 test. In addition, although this study divided individuals into recent and long-term quitters, the exact period of smoking cessation was not ascertained. Further studies are needed to investigate the associations according to the duration of smoking cessation. Third, since the percentages of current smokers (4.1% vs 16%) were significantly different between COVID-19 and non-COVID-19 patients^23^, it is necessary to take this into account in the interpretation of the results of this study. Furthermore, this study period was prior to: 1) the development of effective treatments; 2) a vaccine to reduce disease severity in those infected with SARS-CoV-2; and 3) the emergence of novel variants. All of these are likely to change the severity of disease once infected and have the potential to affect smokers, former smokers, and never smokers differently, particularly if, for example, vaccine uptake and health seeking behavior are very different between these cohorts. Finally, this study included only Korean adults, and further study is warranted to explore the associations in other ethnicities or countries.

## CONCLUSIONS

This study demonstrated that current smokers and recent quitters were associated with an increased risk of death from COVID-19 compared to never smokers. Given the higher possibility of death in smokers who tested positive for SARS-CoV-2 relative to never smoking, smoking is an independent risk factor for COVID-19. Furthermore, long-term quitters were associated with a reduced risk of death from COVID-19 compared with current smokers, suggesting the necessity of continuous smoking cessation among smokers or recent quitters to prevent the fatal risk of COVID-19.

## Data Availability

The data supporting this research cannot be made available for privacy or other reasons. Data may be obtained from a third party and are not publicly available.
